# Circular RNA Expression Profiling Identifies Glaucoma-Related Circular RNAs in Various Chronic Ocular Hypertension Rat Models

**DOI:** 10.3389/fgene.2020.556712

**Published:** 2020-10-07

**Authors:** Xiaoxiao Chen, Rongmei Zhou, Kun Shan, Yanan Sun, Biao Yan, Xinghuai Sun, Jiajian Wang

**Affiliations:** ^1^Department of Ophthalmology and Visual Science, Eye, Ear, Nose and Throat Hospital, Shanghai Medical College of Fudan University, Shanghai, China; ^2^National Health Commission (NHC) Key Laboratory of Myopia, Ministry of Health, Fudan University, Shanghai, China; ^3^Shanghai Key Laboratory of Visual Impairment and Restoration, Fudan University, Shanghai, China; ^4^State Key Laboratory of Medical Neurobiology, Institutes of Brain Science and Collaborative Innovation Center for Brain Science, Fudan University, Shanghai, China

**Keywords:** glaucoma, circular RNA, retinal ganglion cell, intraocular pressure, ocular hypertension, microRNA, TENM4

## Abstract

Circular RNAs are characterized as a class of covalently closed circular RNA transcripts and are associated with a variety of cellular processes and neurological diseases by sponging microRNAs. Expression profiling of circular RNAs in glaucoma, which is a form of optic neuropathy, has not been performed to date. The most common characteristic of all forms of glaucoma is the loss of retinal ganglion cells. While the pathogenesis of glaucoma is not fully understood, intraocular pressure is unquestionably the only proven modifiable factor which makes chronic ocular hypertension (COH) animals the classical glaucoma models. Based on these findings, we completed the first in-depth study of rat retinal circular RNA expression profiling to identify probable biomarkers for the diagnosis of glaucoma. Two ocular hypertension models were induced by episcleral vein ligation (EVL) and microbead injection in rats. Overall, 15,819 circular RNA were detected. Furthermore, 3,502 differentially expressed circular RNAs verified in both COH rats were identified, of which 691 were upregulated and 2,811 were downregulated. Seven significantly downregulated (both log_2_FoldChange < −2.5 and adjusted *P* < 0.001) and seven significantly upregulated (both log_2_FoldChange > 2.5 and adjusted *P* < 0.001) circular RNAs were shown. Six target microRNAs aligned with the top 14 circular RNAs were identified. According to the construction of the circular RNA-microRNA network and circBase information, only RNO_CIRCpedia_1775 had the homologous hsa_circ_0023826 in the human genome. The hsa_circ_0023826 and mRNA of the host gene TENM4 (teneurin transmembrane protein 4) were validated in aqueous humor samples of five glaucoma patients and five cataract control patients. The expression of hsa_circ_0023826 showed a significant decrease in glaucoma patients, while TENM4 mRNA showed no significant difference compared to cataract patients (*P* = 0.024 and *P* = 0.294, respectively). The results of this study comprehensively characterized the expression profiles of circular RNA in glaucoma-affected eyes, as verified by two different ocular hypertension rat models. Together with the target microRNAs underlying the top differentially expressed circular RNAs, a new target of hsa_circ_0023826 and its host gene TENM4 were identified and further verified in the aqueous humor of glaucoma patients, indicating a promising biomarker for the disease.

## Introduction

Glaucoma is a progressive optic neuropathy characterized by optic nerve head damage and visual field defects that ultimately lead to irreversible blindness (Jonas et al., 2017). It is estimated that the number of people diagnosed with glaucoma will increase to 111.8 million in 2040, which has severe socioeconomic implications (Tham et al., 2014). The pathophysiology of glaucomatous neurodegeneration is not fully understood, elevated intraocular pressure (IOP) is the only proven modifiable risk factor for the development and progression of glaucoma characterized by degeneration of retinal ganglion cells (Weinreb and Khaw, 2004). Vision loss, occurring due to the loss of retinal ganglion cells and the degeneration of the optic nerve, has far-reaching effects on independent living and quality of life (Hindle et al., 2019). Currently, IOP-lowering treatment remains the mainstay principle for preserving vision, regardless of the type of glaucoma (Kass et al., 2002). Of the available animal models of glaucoma, the chronic ocular hypertension (COH) rat model serves as a useful tool to be studied in terms of ocular anatomical structures similar to those of humans, short lifespan and well-known genetic information (McKinnon et al., 2009). Considering the long duration of IOP elevation in rats, microbead injection (MBI) and episcleral vein ligation (EVL) have long been the classical techniques to induce animal models of COH (Shareef et al., 1995; Urcola et al., 2006).

Circular RNAs (circRNAs) are a class of non-coding RNAs (ncRNAs) that are abundant during posttranscriptional processes in the brain and eukaryotic organisms (Salmena et al., 2011). Generated through the formation of a covalent bond linking the 3’- and 5’- ends of RNAs by back-splicing, these RNAs are more stable than linear RNAs but also exhibit spatiotemporal properties (Chen et al., 2017). Owing to the ability of circRNAs to sequester microRNAs (miRNAs), especially in the nervous system, circRNAs have pivotal roles in the fine-tuning of posttranscriptional regulation of gene expression in neurological diseases (Lukiw, 2013). Wang et al. revealed that cZNF609 is significantly upregulated in glaucoma-related retinal neurodegeneration. Retinal reactive gliosis and glial cell activation are inhibited by cZNF609 silencing (Wang et al., 2018a). They also found that cZRANB1 is potentially evolved in retinal degeneration induced by COH. cZRANB1 knockdown reduces retinal reactive gliosis which leads to retinal ganglion cells survival (Wang et al., 2018b). Although circRNAs have been reported in some ocular diseases, including diabetic retinopathy, high-throughput sequencing of circRNA expression profiling in glaucoma has not been conducted.

In this study, we hypothesized that circRNAs are dysregulated in COH models. To test this hypothesis, we performed retinal circRNA expression profiling using next-generation sequencing (NGS) in two different COH rat models. From the information obtained by bioinformatics analysis, we searched for the dysregulated circular RNAs and pathways, as well as their target miRNAs and downstream genes involved in COH. Further verification was conducted with aqueous humor samples from glaucoma patients and cataract control patients to verify the circRNAs. Acquisition of the identified circRNAs dysregulated in COH can thus serve as effective biomarkers to predict the progression of glaucoma.

## Materials and Methods

### Animals and Experimental Conditions

All animal experiments were performed in accordance with the National Institutes of Health Guide for the Care and Use of Laboratory Animals (NIH publications No. 8023, revised 1978). This animal study was reviewed and approved by the Eye, Ear, Nose and Throat Hospital of Fudan University. Sprague-Dawley (SD) male rats weighing 180–220 g were maintained under a free-feeding schedule and a 12 h-12 h (light from 6:00 to 18:00 h) light-dark cycle. Rats were randomly distributed into the EVI or MBI group of COH.

### Episcleral Vein Ligation Induced a Chronic Ocular Hypertension Rat Model

The SD rats were used for building the COH model through the ligation of three episcleral veins. In brief, the rats were anesthetized by intraperitoneal injection of xylazine (10 mg/kg) and ketamine (75 mg/kg) mixture before surgery. Throughout the surgery procedure, core body temperature was maintained at 37°C using a thermoregulated heating pad. The conjunctiva and Tenon’s capsule of the left eyes were incised to expose the episcleral veins. Each vein was isolated from the surrounding connective tissue, ligated with 10-0 nylon suture (Alcon Surgical, Fort Worth, TX, United States) and then severed. The sham-operated group received the same surgery without severance and ligation (Urcola et al., 2006). The operated eyes were treated with 0.3% tobramycin (Tobres, Alcon-Couvreur, Puurs, Belgium) after the surgery. IOP was detected at 1, 3, 5, 7, 14, 28, and 60 days after the model was established. The measurement was used by a digital tonometer between 10 a.m. and 2 p.m. to avoid the effect of circadian rhythm (Chen et al., 2011).

### Microbead Injection Induced a Chronic Ocular Hypertension Rat Model

The SD rats were used for building the COH model through the injection of paramagnetic microbeads (Bangs Laboratories, Inc., Fishers, IN, United States; microbead diameter: 10 μm, concentration: 50 mg/mL). In the MBI group, one eye was injected with sterile microbeads, and the sham-operated group was injected with an equivalent volume of phosphate-buffered saline (PBS). After the injection, antibiotic drops of 0.3% tobramycin were placed on each eye. Four weeks after the injection, the second microbead injection was conducted in the abovementioned animals. IOP was detected at 1, 3, 5, 7, 14, 28, and 60 days after the model was established. The measurement was used by a digital tonometer between 10 a.m. and 2 p.m. to avoid the effect of circadian rhythm (Chen et al., 2011).

### CircRNA Identification

#### Isolation of Total RNA

The retinas of three rats from each of two COH (EVL and MBI) groups and the control (Ctrl) group were used to isolate their total RNA by TRIzol (Invitrogen, Carlsbad, CA, United States) and subsequently qualified and quantified by a NanoDrop and Agilent 2100 bioanalyzer (Thermo Fisher Scientific, MA, United States) to determine the total RNA concentration and purity of RNA from each sample. RNA integrity contamination was tested by denaturing agarose gel electrophoresis.

#### Library Construction

The long chain non-coding RNAs (lncRNAs) are RNAs of which length is greater than 200 nt and not involved in protein coding. The lncRNA sequencing library constructed to identify circRNAs have been described in previous studies {Li, 2020 #263}. The first step involved the removal of ribosomal RNA (rRNA) using target-specific oligos and RNase H reagents to deplete both cytoplasmic (5S rRNA, 5.8S rRNA, 18S rRNA and 28S rRNA) and mitochodrial ribosomal RNA (12S rRNA and 16S rRNA) from total RNA preparations (MGIEasy rRNA Depletion Kit, BGI Tech Company, China; Cat. No.: 1000005953). Following SPRI beads purification, the RNA was fragmented into small pieces using divalent cations under elevated temperature. The cleaved RNA fragments were copied into first strand cDNA using reverse transcriptase and random primers, followed by second strand cDNA synthesis using DNA Polymerase I and RNase H. This process removed the RNA template and synthesized a replacement strand, incorporating dUTP in place of dTTP to generate ds cDNA. These cDNA fragments then had the addition of a single “A” base and subsequent ligation of the adapter. After UDG treatment, the incorporation of dUTP quenched the second strand during amplification. The products were enriched with PCR to create the final cDNA library (MGIEasy RNA Directional Library Prep Set, BGI Tec Company, China; Cat. No. 1000006385). The final library was quantitated in two ways: determination of the average molecule length using the Agilent 2100 bioanalyzer instrument and quantification of the library by RT qPCR (TaqMan Probe). The qualified libraries were sequenced pair end using the BGISEQ-500 sequencing platform (BGI Tech Company, China).

#### Prediction of CircRNAs

Bowtie2 v2.2.8 was used to map clean reads to the reference genome (Langmead and Salzberg, 2012). The circRNAs were detected and identified by using CIRI2 and find_circ software packages, separately (Memczak et al., 2013; Gao et al., 2018). We refer to the two software packages to design the basis and pipeline of circRNA identification and optimal choice of parameters, respectively. First, combining the circRNAs both identified by the two softwares, and then intersection of the two identified results based on the location of the circRNAs in the chromosome. Additionally, at least in one group the junction reads in all samples are greater than or equal to 2. Calculating the expression of known and novel circRNAs in each sample, raw counts were first normalized using TPM48 (Zou, 2010). Normalized expression level=(readCount^∗^1,000,000)/the sum of the circRNA read count. The previous reported circRNA annotation was obtained from the database CIRCpedia v2^1^ (Dong et al., 2018).

### Differential Expression Analysis

Differential expression analysis was performed with the DESeq R package (1.10.1). The software offers statistical routines for measuring differentially expressed digital gene data via a model according to the negative binomial distribution. The adjusted *p*-value (*q*-value) and Benjamini and Hochberg false discovery rate were used to provide a balance between the discovery of significant genes and the limitations of false positives (Benjamini and Hochberg, 1995). Differential expression analysis was performed using the *q* < 0.001 and the absolute value of Log2Ratio > 1 as the default threshold to judge the significance of the expression difference (Wang et al., 2010).

### Bioinformatic Analysis

To annotate gene functions, all source genes of differentially expressed circRNAs and downstream target genes were aligned against the Gene Ontology (GO) and Kyoto Encyclopedia of Genes (KEGG) databases. GO enrichment analysis and KEGG enrichment analysis of genes were performed using phyper, a function of R. *Q*-value less than 0.01 was taken as a threshold. GO terms or KEGG terms fulfilling this condition were defined as significantly enriched terms. GO project provided major bioinformatic initiatives to define the constant evolution of our knowledge toward source genes and how they encoded intragenic circRNAs to form products attributed to the cell component, molecular function and biological process. KEGG terms were composed of disease and metabolic pathway enrichment. Prediction terms with q-values less than 0.01 were designated and ranked according to the lowest *q*-values.

### Data Mining Analysis and CircRNA-miRNA-mRNA Network Construction

Significantly differentially expressed circRNAs defined as having an absolute log_2_fold change (FC) value in both MBI/Ctrl and EVL/Ctrl greater than 2.5 and *q*-value less than 0.001 were retained for further analyses. Overall, fourteen highly expressed circRNAs were selected for predicting miRNA-binding sites and downstream mRNAs. Prediction of circRNA-miRNA interactions was performed using the miRanda algorithm for the detection of potential miRNA binding sites in genomic sequences^2^ (Griffiths-Jones et al., 2006). Detected miRNAs with energies less than an energy threshold were selected as potential sites to sponge circRNAs (Enright et al., 2003). Binding-site alignments both passing thresholds and having thermodynamic stability were produced as output. To explore the various target mRNAs of the predicted miRNAs, a protein-protein interaction (PPI) network was constructed to explore the key genes.

### RT-qPCR Validation of CircRNAs and Source Genes

The circRNAs and their source genes highly correlated with COH were selected for RT-qPCR validation. AH was obtained from glaucoma and cataract patients to verify the circRNAs using RT-qPCR (in triplicate). Participants were recruited from June 1, 2019 to June 30, 2019 from the Eye, Ear, Nose and Throat Hospital of Fudan University of Shanghai, China. The study received approval from the Ethical Review Committee of the Eye, Ear, Nose and Throat Hospital and adhered to the tenets of the Declaration of Helsinki. All participants provided written informed consent before the acquisition of aqueous humor. Following TRIzol Reagent (Invitrogen, Carlsbad, CA, United States) manufacturer’s instructions, single-step total RNA extraction was performed on five AH samples in glaucoma and cataract patients. Subsequently, the integrity of RNA was detected by denaturing agarose gel electrophoresis. Total RNA was reverse transcribed to synthesize cDNA, and cDNA was amplified for circRNA and mRNA RT-qPCR analysis using the SuperScript^TM^ IV First-Strand Synthesis System and Powerup^TM^ SYBRTM Green Master Mix (Invitrogen Trading Co., Ltd., Shanghai, China) following the manufacturer’s instructions. cDNA was amplified for the miRNA RT-qPCR analysis using RNase Inhibitor, M-MLV reverse transcriptase, 10 × RT buffer solution (Epicenter, Inc., United States), dNTP Mix, 2.5 mM each (HyTest Ltd.) and RT primers (Bioligo) according to the manufacturer’s instructions. RT-qPCR was performed using a ViiA 7 Dx real-time PCR system (Applied Biosystems) according to the manufacturer’s instructions. Divergent primers (in preference to commonly used convergent primers) were intended for circRNAs and mRNAs chosen for further validation by RT-qPCR. Each circRNA and source gene mRNA were detected in three technical replicates. GAPDH was used as the normalization control for circRNA and mRNA RT-qPCR analysis. Detection of PCR products by insertion of the fluorescent dye SYBR Green. The sense and antisense primers used for GAPDH amplification were 5-GGTGGACCTCATGGCCTACA-3 and 5-CTCTCTTGCTCTCAGTATCCTTGCT-3, respectively.

### Statistical Analysis

Data and results collected were then expressed in terms of the mean and standard deviation. GraphPad Prism Software Version 8.0 (GraphPad Software, La Jolla, CA, United States) and OriginPro Version 8.0 (OriginLab Corporation, Northampton, MA, United States) were used to visualize the data. Significance of RT-qPCR validated circRNA expression differences between the glaucoma and cataract control groups was evaluated by the independent *t*-test using SPSS Statistic Software Version 23.0 (SPSS, Inc., Chicago, IL, United States). *P* < 0.05 are considered to be significant.

## Results

### Intraocular Pressure Changes

**FIGURE 1:**
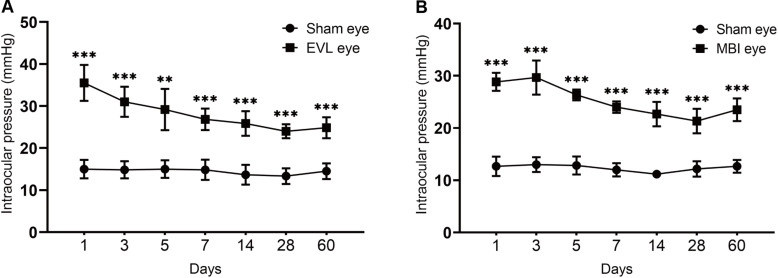
IOP of eyes in the COH groups and sham-operated group. Data are expressed as the mean ± SD. Intraocular pressure, IOP; COH, chronic ocular hypertension; EVL, episcleral vein ligation eyes (*n* = 6); MBI, microbead injection eyes (*n* = 6); sham-operated eyes (*n* = 6). Independent *t*-tests were used to analyze the IOP differences between the COH group and the sham-operated group (*p < 0.05, **p < 0.01, ***p < 0.001). **(A)** EVL-induced COH rat model showed significantly higher IOP than sham eyes at different time points (all *P* < 0.001 except for measurement at 5 days with *P* = 0.003). **(B)** There was a significant difference in IOP between sham and MBI eyes at different time points (all *P* < 0.001).

Changes in IOP over the 60 d after the model was established are shown (Figure 1). The IOP of rats in the COH groups (MBI and EVL) decreased from the peak value during 60 days after the model was established, nevertheless higher than that in the sham-operated group. In the EVL group, the IOP of rats was the highest on the first day (35.50 ± 4.28 vs. 15.00 ± 2.19 mmHg, *P* < 0.001) and remained higher than that of the sham-operated group measured at 3 days (31.00 ± 3.58 vs. 14.83 ± 2.04 mmHg, *P* < 0.001), 5 days (29.17 ± 4.92 vs. 15.01 ± 2.10 mmHg, *P* = 0.003), 7 days (26.83 ± 2.56 vs. 14.83 ± 2.40 mmHg, *P* < 0.001), 14 days (25.83 ± 2.93 vs. 13.67 ± 2.34 mmHg, *P* < 0.001), 28 days (24.00 ± 1.67 vs. 13.33 ± 1.86 mmHg, *P* < 0.001), and 60 days (24.83 ± 2.48 vs. 14.50 ± 1.87 mmHg, *P* < 0.001), respectively (Figure 1A). These results were consistent with the COH model induced by MBI. In the MBI group, IOP at various time points was significantly higher than that of the sham-operated group (all *P* < 0.001, Figure 1B).

### Overview of CircRNA Expression by NGS Identification of Differentially Expressed CircRNAs

**FIGURE 2:**
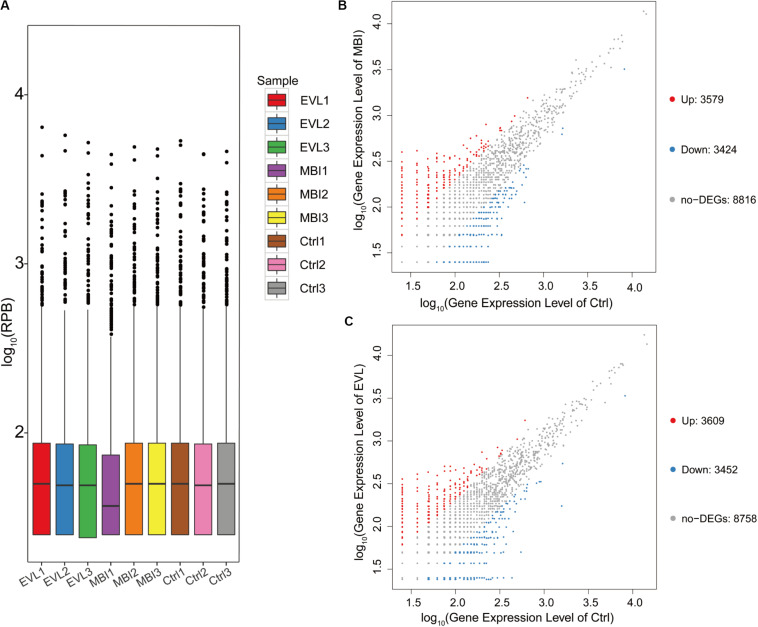
Differential expression of circRNAs in rat retinas. **(A)** Box plot showing the distribution of circRNA expression values in 9 samples from the EVL, MBI and Ctrl groups (EVL, episcleral vein ligation, *n* = 3; MBI, microbead injection, *n* = 3; Ctrl, control, *n* = 3). (RPB, reads per billion) **(B)** Scatter plot of expression level in MBI and Ctrl groups showed differentially expressed circRNAs selected with an absolute log_2_ (fold change) > 1 and *q* < 0.001. **(C)** Scatter plot of expression level in EVL and Ctrl groups showing differentially expressed circRNAs selected with an absolute log_2_ (fold change) > 1 and *q* < 0.001. The red points on the left indicate the upregulated circRNAs, while the blue points indicate the downregulated circRNAs. The gray points in the middle exhibited no significant difference between the COH (chronic ocular hypertension) models and Ctrl group with absolute log_2_(FC) < 1 or *q* > 0.001.

In full, 15,819 circRNAs in three groups of samples were detected by the platform. Distributions of circRNA expression values in the 9 samples of the EVL, MBI, and Ctrl groups after normalization were shown using boxplots (Figure 2A). Scatter plots were used to visualize the expression difference between the MBI and Ctrl groups (Figure 2B). Difference between the EVL and Ctrl groups were shown in Figure 2C. There were 3,579 circRNAs upregulated (log_2_FC > 1 and *q* < 0.001) and 3,424 circRNAs downregulated (log_2_FC < −1 and *q* < 0.001) in the MBI group. Scatter plots of the expression levels in the EVL and Ctrl groups showed 3,609 upregulated and 3.452 downregulated circRNAs.

### Identification of Differentially Expressed CircRNAs and Source Genes

**FIGURE 3:**
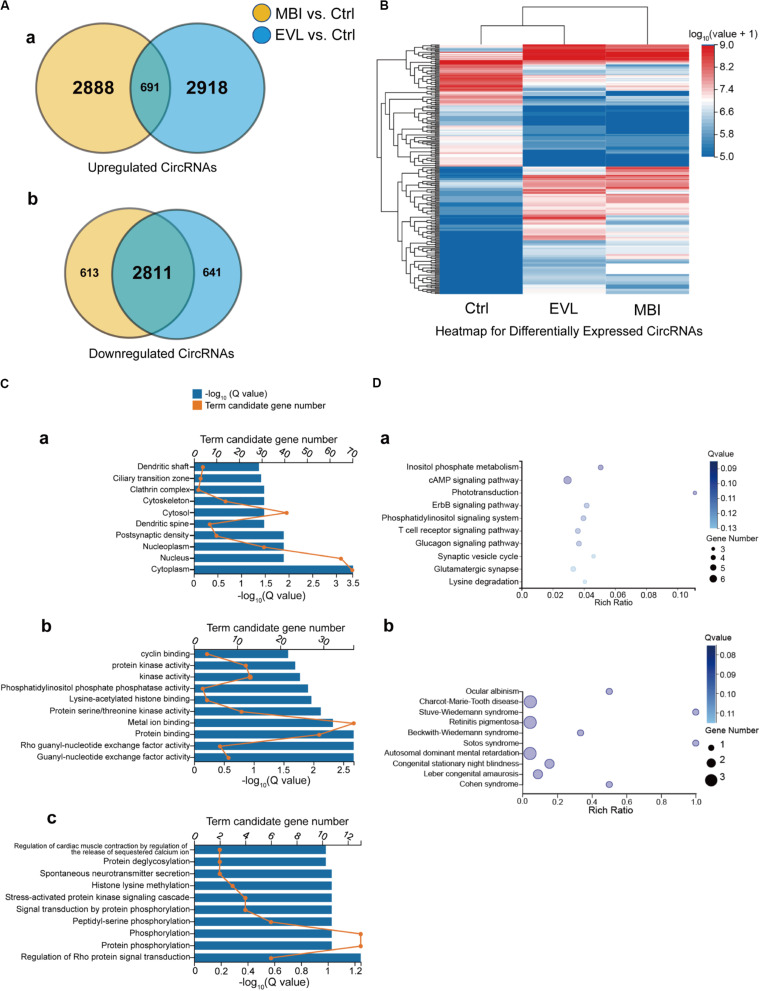
Venn diagram, heatmap, GO and KEGG enrichment results of the differentially expressed circRNAs and their source genes. EVL, episcleral vein ligation; MBI, microbead injection; Ctrl, control; GO, gene ontology; KEGG, Kyoto Encyclopedia of Genes. **(A)** Differentially expressed circRNAs are shown in the two Venn diagrams: **(a)** upregulated circRNAs and **(b)** downregulated circRNAs. Yellow: differentially expressed circRNAs between the MBI and Ctrl groups. Blue: differentially expressed circRNAs between the EVL and Ctrl groups. Intersection: circRNAs differentially expressed with the same tendency in both comparisons (MBI vs. Ctrl, EVL vs. Ctrl). **(B)** Hierarchical clustering of the differentially expressed circRNAs (totality: 237) with expression values above zero were evolved in expression comparison. Expression levels above and below the median expression level are indicated with different colors in all groups. **(C)** 157 intragenic source genes of differentially expressed circRNAs for GO enrichment analysis in terms of cell component **(a)**, molecular function **(b)** and biological process **(c)**. **(D)** KEGG pathway analysis **(a)** and KEGG disease analysis **(b)** for the 157 intragenic source genes.

CircRNA function is related to the source genes that encode circRNAs. Regarding two different methods to induce COH in rats, circRNAs with the same differential expression pattern were evolved in further analysis. There were 691 upregulated circRNAs (Figure 3A-a) and 2,811 downregulated circRNAs (Figure 3A-b) in both the EVL and MBI groups. Heatmap expression profiles included the circRNAs screened out by Venn diagram defined as the same expression pattern in two COH models and expression values above zero. Finally, 237 circRNAs were selected to visualize the expression value differences among the three groups (Figure 3B). The top 10 general GO terms were listed in all comparison groups categorized by cell component, molecular function and biological process ranked by their *q*-values. The results indicated that most augmented and statistical different cell component term was cytoplasm (Figure 3C-a). In terms of molecular function, gunny-nucleotide exchange factor activity, Rho gunny-nucleotide exchange factor activity and protein biding were the major different terms (Figure 3C-b). In terms of biological process, no significantly statistical different term was found with a *q*-value greater than 0.01 (Figure 3C-c). Furthermore, KEGG pathway enrichment analysis was performed, and significantly enriched pathways were not discovered (all *Q* > 0.01).

### Construction of the CircRNA-miRNA-mRNA Coexpression Network

Among the differentially expressed circRNAs, the highest significantly differentially expressed 14 circRNAs were shown in Table 1 (absolute log_2_FC_EVL/Ctrl > 2.5, absolute log_2_FC_MBI/Ctrl > 2.5, both *Q* < 0.001). Then, six of the predicted responses to the top 14 circRNAs by miRanda and verified by NGS (absolute log_2_FC_EVL/Ctrl > 1, absolute log_2_FC_MBI/Ctrl > 1, both *Q* < 0.001) were shown in Table 2. Next, we predicted the target genes of these six miRNAs using TargetScan, RNAhybrid, and miRanda. We generally accepted that the results overlapped among these three databases and found targeted 295 mRNAs.

**TABLE 1:** Biological information regarding the highest upregulated and downregulated circRNAs.

CIRCpedia_ID/circRNA_ID	log_2_FC_EVL/Ctrl	*Q*-value	log_2_FC_MBI/Ctrl	*Q*-value	Chromosome	Best transcript	Gene symbol
**Upregulation**							
RNO_CIRCpedia_3860	2.930908846	7.05E-33	2.503896293	1.32E-20	Chr2	NM_021763	*Arfip1*
RNO_CIRCpedia_2508	3.022651415	7.08E-36	2.745714213	3.20E-26	Chr6	ENSRNOT00000008687.5	*Memo1*
RNO_CIRCpedia_4101	2.880613293	2.49E-45	2.800268966	5.87E-40	Chr9	NM_001109968	*Gls*
RNO_CIRCpedia_9044	2.511005592	8.79E-22	2.503896293	1.32E-20	Chr10	ENSRNOT00000004740.5	*Rufy1*
Chr1:143143054| 143143524	3.568338767	3.96E-59	4.04248883	4.15E-85	Chr1	/	*Pde8a*
Chr5:106202419| 106208769	2.632469104	1.31E-24	2.6346829	1.82E-23	Ch5	/	*Mllt3*
Chr2:170842069| 170843772	2.613263925	1.57E-56	2.684238725	2.11E-58	Chr2	/	*/*
**Downregulation**							
RNO_CIRCpedia_1920	–3.12498091	3.98E-38	–3.02066893	2.09E-35	Chr1	NM_001100975	*Vps13a*
RNO_CIRCpedia_389	–2.62018876	1.74E-23	–2.51587678	1.67E-21	Chr11	NM_031615	*Zfp148*
RNO_CIRCpedia_3802	–2.6887976	3.15E-24	–2.52559193	1.01E-21	Chr2	ENSRNOT00000075947.2	*RGD1307100*
RNO_CIRCpedia_2544	–2.62990392	1.07E-23	–2.52559193	1.01E-21	Chr6	ENSRNOT00000009779.7	*Nbas*
RNO_CIRCpedia_2623	–2.85367889	1.72E-29	–2.74936691	3.56E-27	Chr6	NM_020083	*Ralgapa1*
RNO_CIRCpedia_1775	–2.6887976	3.15E-24	–2.52559193	1.01E-21	Chr1	ENSRNOT00000015181.6	*Tenm4*
chr5:148050938| 148057784	–2.62990392	1.07E-23	–2.52559193	1.01E-21	Ch5	/	*Mllt3*

*CIRCpedia v2 (https://www.picb.ac.cn/rnomics/circpedia/); FC, fold change; EVL, episcleral vein ligation; Ctrl, control; Q-value, adjusted p-value; MBI, microbeads injection.*

**TABLE 2:** Predicted miRNA response elements regarding the circRNAs and validated by next-generation sequencing.

miRNA_ID	CircRNA	Log2FC_EVL/Ctrl	*Q*-value	Log2FC_MBI/Ctrl	*Q*-value
rno-miR-183-5p	RNO_CIRCpedia_2508	–1.795	<0.001	–1.96385	<0.001
rno-miR-130b-3p	RNO_CIRCpedia_4101	–2.41225	<0.001	–1.73219	<0.001
rno-miR-146a-5p	RNO_CIRCpedia_389	1.25109	<0.001	1.34687	<0.001
	RNO_CIRCpedia_1775				
rno-miR-3543	RNO_CIRCpedia_389	1.02421	<0.001	1.4054	<0.001
	RNO_CIRCpedia_2544				
	RNO_CIRCpedia_1775				
rno-miR-3553	RNO_CIRCpedia_1775	3.12842	<0.001	3.27945	<0.001
rno-miR-539-5p	RNO_CIRCpedia_1775	7.84239	<0.001	8.16942	<0.001

*CIRCpedia v2 (https://www.picb.ac.cn/rnomics/circpedia/); FC, fold change; EVL, episcleral vein ligation; Ctrl, control; Q-value, adjusted p-value; MBI, microbeads injection.*

**FIGURE 4:**
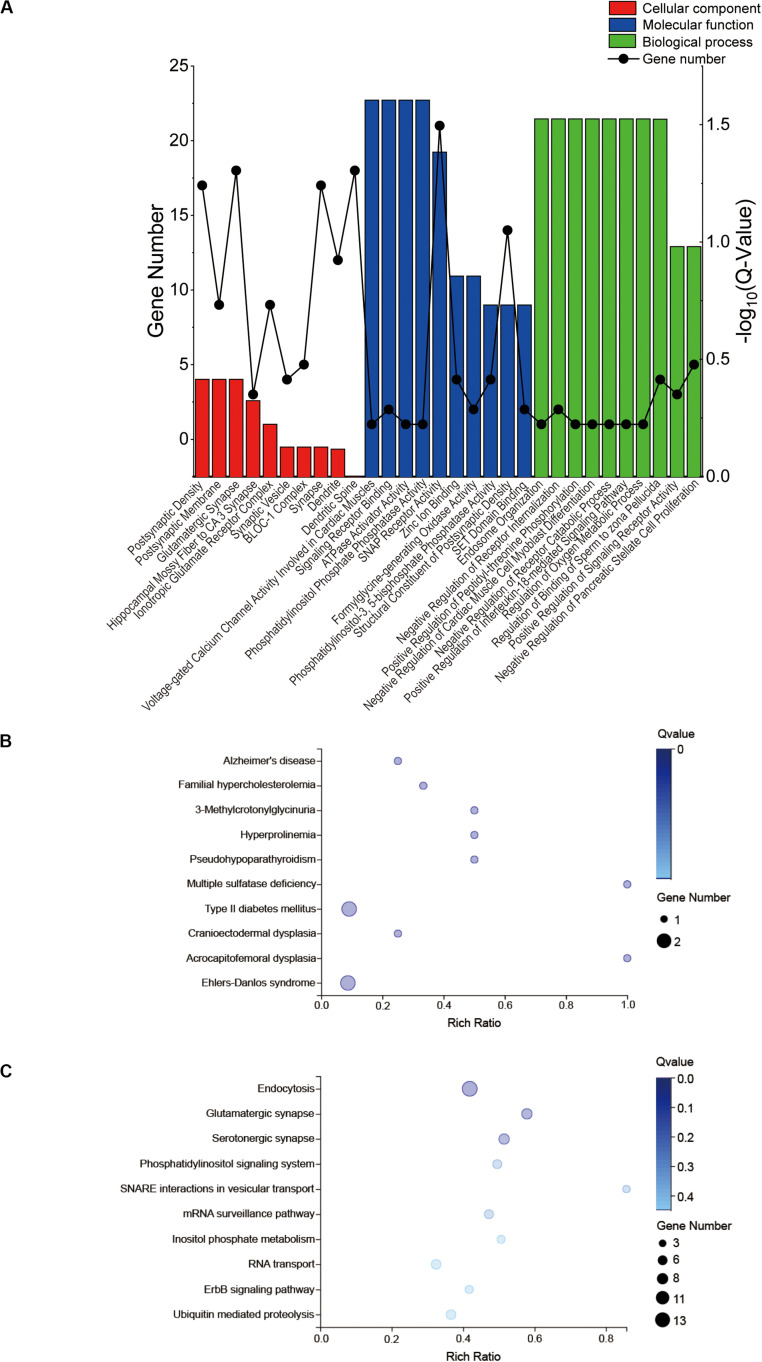
GO and KEGG enrichment results of the target genes. GO, gene ontology; KEGG, Kyoto Encyclopedia of Genes. **(A)** A total of 295 target genes of six differentially expressed miRNAs responded to the top 14 circRNAs for GO enrichment analysis in terms of cell component, molecular function and biological process. **(B)** KEGG disease analysis **(C)** and KEGG pathway analysis for the target genes.

**FIGURE 5:**
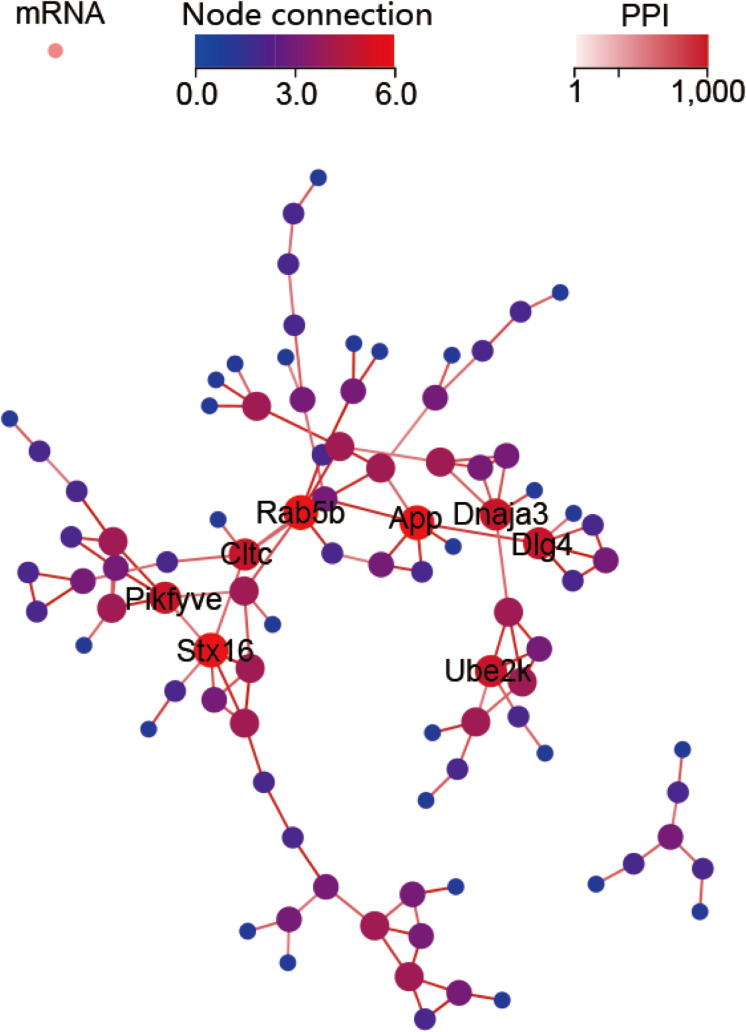
Protein-protein interaction (PPI) network for the target mRNAs. Target genes with five or six node connections are labeled with gene symbols.

The GO profiles provided major bioinformatics initiatives to define the continuing evolution of our knowledge regarding target genes and their contributions to cell component, molecular function and biological process. Prediction terms with *q*-values less than 0.01 were designated. The top 10 GO terms with the lowest *q*-values were listed in all comparison groups categorized by cell component, molecular function and biological process ranked by their *q*-values. Our results indicated that postsynaptic density, postsynaptic membrane and glutamatergic synapse were significantly different in cell component terms (all *Q* > 0.01). In terms of molecular function, voltage-gated calcium channel activity involved in cardiac muscles, signaling receptor binding, ATPase activator activity and phosphatidylinositol phosphate phosphatase activity were the major different functions of molecules. In terms of biological process, endosome organization was the most obvious biological process (Figure 4A). The most highly enriched disease according to the *q*-values and gene numbers was type 2 diabetes mellitus (T2DM) (Figure 4B). Furthermore, KEGG pathway enrichment analysis was performed, and its significantly enriched pathways were then selected and ranked according to their *q*-values, with endocytosis being the most enriched (Figure 4C). According to the PPI network profile, mRNAs with more than 4 connection nodes were Rab5b (member RAS oncogene family), App (amyloid beta precursor protein), Stx16 (syntaxin 16), Pikfyve (phosphoinositide kinase, FYVE-type zinc finger), Dnaja3 (DnaJ heat shock protein family member A3), Cltc (clathrin heavy chain) and Dlg4 (discs large MAGUK scaffold protein 4) (Figure 5).

### Verification of NGS Data by RT qPCR

**FIGURE 6:**
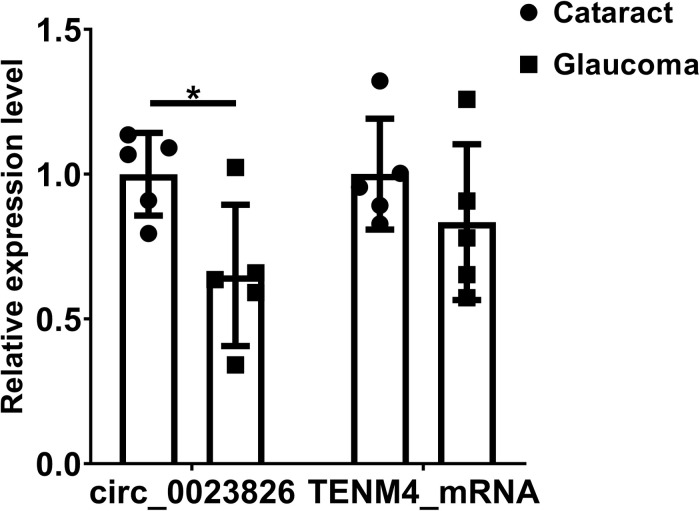
Validation of circ_0023826 expression in clinical samples. GAPDH was used as the reference gene to compare the expression difference of hsa_circ_0023826 and the level of Tenm4 mRNA between glaucoma and cataract patients’ aqueous humor. *P < 0.05.

**FIGURE 7:**
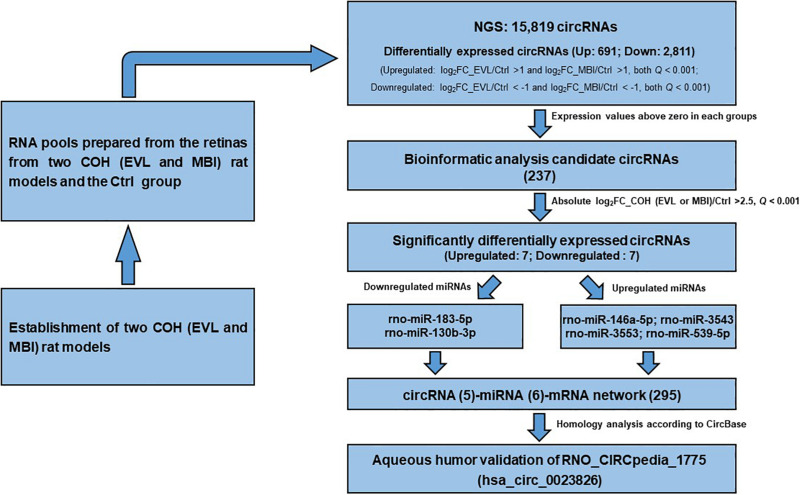
Flow chart of identification of has_circ_0023826. COH, chronic ocular hypertension; EVL, episcleral vein ligation; MBI, microbeads injection; Ctrl, control; circRNA, circular RNA; FC, fold change; miRNA, microRNA.

Considering that most of circRNAs were highly conserved in mammals, target circRNAs, including RNO_CIRCpedia_2508, RNO_CIRCpedia_4101, RNO_CIRCpedia_389, RNO_CIRCpedia_1775, and RNO_CIRCpedia_2544, were evolved in corresponding to human circRNAs by CircBase^3^. Only RNO_CIRCpedia_1775 had a homologous source gene—teneurin transmembrane protein 4 (TENM4) in human genome, which encoded hsa_circ_0023826. To reassess the possible differences by NGS, hsa_circ_0023826 was analyzed by RT-qPCR in five glaucoma AH samples and five cataract AH samples in patients. The clinical characteristics of these patients were shown in Table 3. The results detected by RT-qPCR was in keeping with the sequencing results (Figure 6). The flow chart described the steps that how the hsa_circ_0023826 was identified (Figure 7).

**TABLE 3:** Clinical features of cataract and glaucoma patients.

Patient_ID	Diagnosis	Age	Gender	Gender of eye	IOP
1	Cataract	59	Female	Left	15
2	Cataract	55	Female	Right	14
3	Cataract	68	Male	Right	19
4	Cataract	62	Female	Right	13
5	Cataract	61	Male	Right	13
6	Glaucoma	45	Male	Left	35
7	Glaucoma	52	male	Right	29
8	Glaucoma	52	Female	Left	32
9	Glaucoma	49	Male	Left	35
10	Glaucoma	70	Female	Right	38

*IOP, intraocular pressure.*

## Discussion

CircRNAs are ubiquitous ncRNAs that are more stable and highly conserved transcripts compared with other linear lncRNAs (Piwecka et al., 2017). These RNAs are stable and always enriched in some specific tissues or biological processes (Filipowicz et al., 2008). These properties make circRNAs appealing biomarkers. CircRNAs have been reported to be involved in the pathogenesis of many ocular diseases, such as cataracts, corneal vascularization, diabetic retinopathy and neurodegenerative diseases (Luo et al., 2017; Shan et al., 2017; Zhang et al., 2017; Wang et al., 2018a, b; Wu et al., 2020). However, the role of circRNAs in glaucoma has not been fully elucidated. In our study, NGS was used to find the expression difference of circRNAs between the two different COH rat models induced by EVL and MBI, together with the Ctrl group to pursue more reliable differentially expressed circRNAs. In total, 15,819 circRNAs were identified using sequencing, and the following analysis provides a thorough evaluation of the genetic mechanism underlying glaucoma. Seven significantly upregulated and seven significantly downregulated circRNAs were further evaluated, and six aligned miRNAs together with downstream target mRNAs were found to construct the circRNA-miRNA network. Only RNO_CIRCpedia_1775 had a homologous source gene of TENM4 in the human genome, which encodes hsa_circ_0023826. The hsa_circ_0023826 and mRNA of Tenm4 were further validated in the AH of glaucoma compared to cataract patients, which was largely consistent with the NGS results.

To the best of our knowledge, this report is the first to describe circRNA analysis in ocular diseases by using two different animal models to verify the reliability and universality of the conclusions. From the pathophysiological point of view, IOP is the primary risk factor for glaucoma (Jonas et al., 2017). The classical animal models established to induce glaucoma increase the IOP to form COH. To pursue the more reliable differentially expressed circRNAs, two COH rat models were induced by EVL and MBI. Ruiz and colleagues showed that elevated IOP persisted for approximately 4 weeks, which corresponded to our results of IOP normalizing to between 20 and 30 mmHg after approximately 8 weeks (Ruiz-Ederra and Verkman, 2006). The IOP in MBI group remained above 21 mmHg for 8 weeks due to reinjection at 4 weeks according to the method. EVL is one of the earliest rat models to induce COH with the advantage of not causing profound ocular complications (Shareef et al., 1995). In addition, retinal ganglion cells death in EVL model was directly correlated with the increase level of IOP and the duration of the model established (Garcia-Valenzuela et al., 1995). Injection of microbeads could also increase the IOP by occlusion of trabecular meshwork, while possible side effects, such as inflammation, might be caused by the internal canal of the injection syringe (Chen et al., 2011). Therefore, application of two different animal models could minimize the interference caused by the trauma in animal modeling, which supported our differentially expressed circRNAs observed in COH rats.

In our study, differentially expressed circRNAs were identified revealing significant differences between the COH (EVL or MBI) and Ctrl groups and suggesting their roles in glaucoma pathogenesis. Concerning the top seven upregulated and seven downregulated circRNAs, the expression values manifested major differences with absolute log_2_FC more than 2.5 in both COH groups. The circRNAs could regulate target genes expression mostly by miRNA sponges, as well as RNA-binding protein repossessing mediation or regulators of transcription (Chen, 2016; Salzman, 2016). Titration of miRNAs was characterized by opposite expression patterns in circRNAs and sponged miRNAs. Afterward, six circRNA-miRNAs predicted by binding sites and validated by the differential expression pattern were observed in our study (Guo et al., 2019). Enrichment of downstream target genes in KEGG showed that the most obvious pathway was the cAMP signaling pathway. Previously, adenosine has been reported to exert the neuroprotective effects of neurons and as the possible treatment site for central nervous system disorders (Goldberg et al., 1988; Ahmad et al., 2014). Physiologically, binding of the adenosine and receptors stimulate adenylyl cyclase (AC) and increase cAMP levels (van Calker et al., 1979). Some of the receptors in the inner retina layer provided the sites for modulators to affect the retinal ganglion cell functions and IOP fluctuation (Oku et al., 2004; Avni et al., 2010). The receptor exemplified as A_3_ is evolved in neurite outgrowth-promoting effects of adenosine and provides a new tool to make anti-glaucoma medications (Nakashima et al., 2018).

To characterize the target genes that eventually influence the pathogenesis of glaucoma, a PPI network was established to show the key genes. Key target genes were defined with more than four connection nodes. In the target gene KEGG disease enrichment analysis, Alzheimer’s disease (AD) showed the lowest *q*-value. Evolved gene–App centered in the network has been indicated to change in degenerative retina. App mRNA was regulated by downregulated expressed rno-miR-183-5p and rno-miR-130b-3p. Translation products of App therefore are predicted to increase in retina. Studies of retinas in postmortem subjects clearly found the increase of App accumulation in those diagnosed as AD (den Haan et al., 2018). Proteolysis of App produces amyloid-β accumulation, which is the typical pathological change of AD. Visual dysfunction is commonly found in AD patients and correlated with severity of the disease, even in preclinical patients with no obvious intellectual disabilities (O’Bryhim et al., 2018). This correlation provides a novel target to inhibit the pathogenesis of neurodegenerative disorders.

Considering that the results were based on NGS in rats, the homologous circRNAs must be identified in the human genome for the treatment of glaucoma. By homogeneous comparisons between rats and human beings, RNO_CIRCpedia_1775 isogenous hsa_circ_0023826 was found decreased in the AH samples of glaucoma patients compared to cataract controls, while mRNA of TENM4 remained invariable. Mutations of TENM4 in humans were manifested as an essential tremor, which is a common movement disorder (Hor et al., 2015). The symptoms of tremor could be partly explained by abnormal TENM4 expression leading to skeletal regeneration problems (Yamaguchi et al., 2012). Tenm4 plays a strong role in oligodendrocyte differentiation and myelination of small diameter axons (Suzuki et al., 2012). Combined with no difference found in AH samples between glaucoma and cataract patients, we should further study the role of has_circ_0023826 encoded by TENM4 as an interference site for glaucoma.

The current study has several limitations including small sample size of three retinas in each group. Besides, more studies about retina ganglion cell functions will be required to determine the exact devotion of hsa_circ_0023826 for the pathogenesis of glaucoma. IOP is determined by the balance between secretion and drainage of AH. AH is secreted by the ciliary body and exits mostly through the trabecular meshwork. Resistance to aqueous outflow through the trabecular meshwork is increased in glaucoma especially in the type of primary open-angle glaucoma which leads to elevated IOP. Therefore, trabecular meshwork and AH are also appropriate targets to study the mechanism of glaucoma. We used AH to validate the role of hsa_circ_0023826 in glaucoma. Trabecular meshwork is also further target for the group to study.

## Conclusion

This report describes the first comprehensive study of glaucoma-related circRNA expression profiles identified by two different glaucoma animal models. The application of NGS helped us to identify glaucoma-related circRNAs. The RNO_CIRCpedia_1775 isogenous hsa_circ_0023826 in the human genome together with the source gene TENM4 was further validated in the aqueous humor of glaucoma patients. Moreover, we identified Rab5b, App, Stx16, Pikfyve, Dnaja3, Cltc and Dlg4 as key genes impacting glaucoma pathogenesis by transcription differences. Finally, hsa_circ_0023826 was identified as a biomarker for the diagnosis of glaucoma; this circRNA is encoded by TENM4 and targets miR-146a-5p, miR-3543, miR-3553, and miR-539-5p.

## Data Availability Statement

The transcriptomics datasets generated during the current study have been registered with the SRA database and are accessible via SRA ID PRJNA656441.

## Ethics Statement

The studies involving human participants were reviewed and approved by the Ethical Review Committee of the Eye, Ear, Nose and Throat Hospital. The patients/participants provided their written informed consent to participate in this study. The animal study was reviewed and approved by the Ethical Review Committee of the Eye, Ear, Nose and Throat Hospital.

## Author Contributions

XC, JW, and XS conceived and designed the experiments. XC, RZ, YS, and KS performed the experiments. XC analyzed the data. XC, RZ, BY, and XS wrote the manuscript. All authors contributed to the article and approved the submitted version.

## Conflict of Interest

The authors declare that the research was conducted in the absence of any commercial or financial relationships that could be construed as a potential conflict of interest.

## Footnotes

1 https://www.picb.ac.cn/rnomics/circpedia/

2 https://cbio.mskcc.org/microrna_data/manual.html

3 http://www.circbase.org/
